# Processes for evidence summarization for patient decision aids: A Delphi consensus study

**DOI:** 10.1111/hex.13244

**Published:** 2021-05-15

**Authors:** Peter Scalia, Catherine H Saunders, Michelle Dannenberg, Anik MC Giguere, Brian S Alper, Tammy Hoffmann, Lilisbeth Perestelo‐Perez, Marie‐Anne Durand, Glyn Elwyn

**Affiliations:** ^1^ The Dartmouth Institute for Health Policy and Clinical Practice Geisel School of Medicine at Dartmouth College Lebanon NH USA; ^2^ Faculte de medicine Universite Laval Quebec City Quebec Canada; ^3^ Computable Publishing LLC Ipswich MA USA; ^4^ Institute of Evidence‐Based Healthcare Bond University Gold Coast Qld Australia; ^5^ Evaluation Unit of the Canary Islands Health Service. REDISSEC Tenerife Spain

**Keywords:** Delphi, evidence summarization, evidence‐based medicine, patient decision aids, shared decision making

## Abstract

**Background:**

Patient decision aids (PDAs) should provide evidence‐based information so patients can make informed decisions. Yet, PDA developers do not have an agreed‐upon process to select, synthesize and present evidence in PDAs.

**Objective:**

To reach the consensus on an evidence summarization process for PDAs.

**Design:**

A two‐round modified Delphi survey.

**Setting and participants:**

A group of international experts in PDA development invited developers, scientific networks, patient groups and listservs to complete Delphi surveys.

**Data collection:**

We emailed participants the study description and a link to the online survey. Participants were asked to rate each potential criterion (omit, possible, desirable, essential) and provide qualitative feedback.

**Analysis:**

Criteria in each round were retained if rated by >80% of participants as desirable or essential. If two or more participants suggested rewording, reordering or merging, the steering group considered the suggestion.

**Results:**

Following two Delphi survey rounds, the evidence summarization process included defining the decision, reporting the processes and policies of the evidence summarization process, assembling the editorial team and managing (collect, manage, report) their conflicts of interest, conducting a systematic search, selecting and appraising the evidence, presenting the harms and benefits in plain language, and describing the method of seeking external review and the plan for updating the evidence (search, selection and appraisal of new evidence).

**Conclusion:**

A multidisciplinary stakeholder group reached consensus on an evidence summarization process to guide the creation of high‐quality PDAs.

**Patient contribution:**

A patient partner was part of the steering group and involved in the development of the Delphi survey.

## 1 BACKGROUND

Patient decision aids (PDAs) are tools that help patients and their clinicians make preference‐sensitive decisions together.^1^ They are typically defined as ‘evidence‐based tools designed to help patients make specific and deliberated choices among healthcare options.^1^ PDAs supplement (rather than replace) clinicians' counseling about options’.^1^, ^2^ They promote patient engagement in health decision making and collaboration between patients and their care team, increase patient knowledge and align patients’ choices with their preferences.^1^ Further, PDAs are being increasingly designed with, and for, patients with lower health literacy skills. For example, Option Grid PDAs are written at a sixth‐grade reading level, and some versions include pictures that have been user‐tested with low literacy patients to improve their understanding and recall of the health information.^3^, ^4^, ^5^, ^6^, ^7^ Therefore, the information included in PDAs can substantially affect patients' decisions. For this reason, patients and clinicians expect the information in PDAs to be evidence‐based and rigorously selected and summarized.

However, the approach that PDA developers use to select and summarize the evidence in PDAs is inconsistent and often incomplete or not described.^2^ A recent international survey of 15 PDA developers confirms that they do not have an agreed‐upon, standardized process to select and summarize evidence.^8^ They did not always document the evidence selection and summarization process.^8^ Most organizations reported using existing systematic reviews and clinical practice guidelines to select and summarize information for PDAs.^8^ Less than half reported using a standard, documented approach to guide the evidence selection and summarization.^8^ When the approach was documented, the documents offered varying levels of detail.^8^ Common evidence summarization steps identified were as follows: tool‐relevant question formation, search strategies, evidence appraisals and updating policies.^8^ There was no standardized process across organizations to summarize evidence for PDAs.^8^

The International Patient Decision Aids Standards (IPDAS) Collaboration developed criteria for assessing the quality of PDAs.^9^ These criteria are also used by PDA producers to guide the development of the aids. However, only five items of the IPDAS checklist cover the selection and summarization of evidence, and none provide guidance about recommended methods for evidence selection and summarization for PDAs.^9^ A 2013 review of the literature conducted by the IPDAS working group on the synthesis of scientific evidence highlighted the importance of rigorously selecting and summarizing evidence used to populate a PDA. They did not provide clear practical guidance on how to conduct evidence summarization for the development of PDAs, except recommending that developers apply the Grading of Recommendations Assessment, Development and Evaluation (GRADE) methodology.^10^ Furthermore, the IPDAS instrument and the IPDAS minimum standards do not offer additional information or guidance on the steps required to select and summarize evidence‐based information for PDAs.^11^, ^12^ As part of a recent update of IPDAS, researchers analysed the evidence characteristics of 471 publicly available PDAs, and only 14% of them reported ‘at least one of the steps used to select the evidence included in the decision aid, such as how it was searched for, appraised, or summarized’.^13^ Other efforts to evaluate or certify the quality of PDAs have emerged,^14^ but none of those standards or certification bodies describe recommended methods and criteria that PDA producers should follow when selecting and summarizing evidence for patient‐facing tools.

Although consensual and validated methods for evidence summarization exist for other evidence resources, such as clinical practice guidelines, there is no agreed‐upon process for the selection and summarization of evidence for PDAs. Evidence summarization processes for other resources have become increasingly standardized, which promotes transparency and rigour, while minimizing the risk of bias in the end product.^2^, ^23^ The same level of scrutiny is justified when developing PDAs, as they may directly influence patient care and decision making. The selection and identification of patient‐relevant outcomes, analysis of patient concerns and priorities, description of the quality of evidence and communication of uncertainty in ways that patients understand warrant the development of an agreed‐upon process and related steps and criteria that are specific to PDAs. The target audience, scope and content differ substantially enough from clinical practice guidelines to require a tailored evidence summarization process. Additionally, the IPDAS Collaboration imposes some prerequisites on the evidence summarization process on which the PDA will be based. For example, the IPDAS Collaboration requires that developers summarize the evidence regarding all health options available to a patient facing a specific health problem, with positive and negative features of each option presented with equal amounts of details.^24^ Efforts to develop an agreed‐upon evidence summarization process for PDAs should incorporate the substantial body of related evidence summarization guidance previously developed by other groups.^17^

The aim of the study was to reach consensus, using a modified Delphi survey, on a process, related steps and criteria for selecting and summarizing evidence for PDAs. This will, in turn, assist PDA developers in improving the transparency and rigour, while minimizing the risk of bias in the evidence summarization processes used in PDA development.

## 2 METHODS

### 2.1 Design

Our study protocol was published, and a summary is presented here.^25^ The modified Delphi method has been previously used in the development of a quality criteria framework for PDAs.^2^, ^26^ We used this method because it is the most practical and scalable to obtain feedback from a large number of stakeholders in different geographical locations. We predetermined that there would be two Delphi rounds, with a third if needed. The anonymous responses from participants in the first round were fed back to them in the second round. Throughout the process, a panel of relevant stakeholders in the field of PDA development provided feedback about the evolving set of evidence summarization steps and criteria. Ethical approval was received from the Committee for the Protection of Human Subjects (STUDY00031042).

### 2.2 Participants

To maximize the generalizability and applicability of the criteria, we invited the following groups to complete the surveys: (1) the Global Inventory of Patient Decision Support Developers, which includes all known developers of PDAs who created or updated a tool within the last five calendar years (the inventory was last updated 4 October 2019), (2) all members of the IPDAS group, (3) the shared decision‐making listserv, (4) the Society for Participatory Medicine listserv, (5) an overdiagnosis Google Group, (6) the evidence‐based health‐care listserv, (7) the Society for Medical Decision Making, (8) the Society of Behavioural Medicine (Health Decision Making Interest Group), (9) the HTAi‐ISG patient involvement listserv, (10) the GRADE working group, (11) the Guidelines International Network, (12) a convenience sample of policymakers with interest and expertise in PDA certification, (13) the BMJ patient group and (14) the ProPublica Patient Safety Community. Membership in one of the groups listed above was the only criterion for inclusion in our study.

### 2.3 Steering group

We convened a steering group to oversee the project and make strategic decisions about the study design, data collection and analysis processes, as well as agree on a final set of steps and criteria. The steering group also generated an initial set of criteria for the Delphi process and managed the distribution of the survey.

An invitation to join the steering group was posted in the Shared@Shared Decision Making Network Facebook group (745 members) on 30 June 2017. The post invited all members in the Facebook group to join an in‐person meeting about evidence summarization during the International Shared Decision Making (ISDM) conference held in Lyon, France, between 2 and 5 July 2017. Following the conference, a summary of the meeting was posted on Facebook for those who expressed interest in evidence summarization of PDAs but could not attend.

The steering group convened by videoconference in September 2017. It included 9 international experts in the areas of PDA development, evaluation and implementation, evidence summarization and clinical practice guidelines. The experts were based in the United States (n = 6), Canada (n = 1), Australia (n = 1) and Spain (n = 1), all of whom are authors of this study. The steering group also included 1 patient representative. To avoid contaminating Delphi results or duplicating their views, the steering group members unanimously decided not to complete the Delphi surveys.

### 2.4 Survey development

At the first meeting of the ISDM conference in Lyon, France, the steering group developed a spreadsheet that detailed the evidence summarization process inherent to PDA development. The first draft of the process included 18 criteria. The 18 criteria were combined with the 8 existing standards for the summarization of clinical practice guidelines as outlined by the National Academy of Medicine and US Preventive Services Task Force Standards.^27^ These 26 criteria made up the first draft of the proposed evidence summarization process and steps. The draft was embedded in a Google Document and shared with the members of the steering group so they could iteratively refine and finalize the process. Overall, this work produced three separate iterations of the evidence summarization process with slightly different wording and grouping of steps and criteria. Each iteration was reviewed and discussed by the steering group members. After no additional changes were suggested, four chronological phases of evidence summarization, consisting of 48 criteria categorized into 13 steps, made up the first survey round in April 2018 (see Appendix S1).

### 2.5 Data collection

#### 2.5.1 Round 1 survey

The round 1 survey invitation (see Appendix S2) was sent by email and provided a brief outline of the study and the link to the online survey. Consent was inferred by participants’ completion of the survey. The first page of the survey was a brief participant information sheet. Following the information sheet, participants were asked to complete demographic questions and provide their email address so that they could be contacted for round 2 of the survey. Next, participants were asked to provide their input on the phases, steps and criteria (including inclusion, wording, grouping, order and any other comments). Specifically, they indicated whether each criterion should be omitted, possibly kept, or whether they considered the criterion to be essential or desirable to the process using a 4‐point Likert scale (omit, possible, desirable and essential). Participants were also given the opportunity to provide rewording suggestions, suggest additional phases, steps or criteria or provide additional comments or questions. Participants were not required to provide qualitative feedback but had to select a response on the Likert scale for each criterion to progress through the survey. Participants could exit the survey at any time. The survey was open from 16 July to 1 September 2018. During that time period, two automated email reminders were sent to participants to complete the survey.

#### 2.5.2 Round 2 survey

Round 1 participants were invited to complete the round 2 survey via email. The round 2 survey included a summary of the round 1 results: the percentage of participants who thought each criterion should be retained or excluded and the changes made based on the qualitative feedback. Participants were invited to indicate whether each criterion should be omitted, possibly kept, or whether they considered the criterion to be essential or desirable to the process using the same Likert scale as round 1. They could also provide additional rewording suggestions, comments or questions for criteria that did not reach consensus in round 1 and new criteria proposed by participants during the first round. The survey was available from 24 April to 31 May 2019. During that time frame, two automated email reminders were sent to participants to complete the survey.

### 2.6 Data analysis

Following round 1, the ratings were summarized using percentages. If >80% of participants rated a criterion in the lower two categories (omit, possible) or in the higher two categories (desirable and essential), we considered consensus as having been reached and the criterion was removed or retained accordingly. Following the round 1 survey, a consensus meeting involving the steering group was held. The steering group reviewed and discussed the ratings and qualitative feedback, including rewording suggestions, suggestions to add new phases, steps or criteria, and more general comments or questions. The wording or order of the phases, steps or criteria were revised if two or more respondents suggested it or if the steering group members agreed that the phase, step or criterion would benefit from rewording, reordering or merging. The same process was conducted following the round 2 survey.

Only fully completed surveys were included in the analysis. Based on the round 2 results and feedback, the steering committee deemed it unnecessary to conduct a third Delphi round. This method to determine the number of survey rounds was implemented successfully in the past to develop a measure of organizational readiness for patient engagement.^28^

## 3 RESULTS

### 3.1 Participants

In the first Delphi round, 50% (n = 131/260) of participants who started the survey completed it. The majority, 58% (n = 76), of respondents were female. Overall, 26 countries were represented (see Table 1 for details). A total of 49% (64/131) of participants selected multiple roles (see Appendix S3 to view the roles of participants in the first Delphi round).

**TABLE 1:** Participant characteristics

Characteristic	Round 1 (n = 131)	Round 2 (n = 114)
Gender		
Male, n (%)	54 (41)	47 (41)
Female, n (%)	76 (58)	67 (59)
Other, n (%)	1 (0)	0 (0)
Country		
United States, n (%)	52 (40)	50 (44)
United Kingdom, n (%)	16 (12)	17 (15)
Australia, n (%)	6 (5)	7 (6)
Canada, n (%)	9 (7)	6 (5)
Other*, n (%)	48 (37)	34 (30)
Ethnicity		
White, n (%)	113 (87)	95 (83)
Hispanic, Latino/a or Spanish origin, n (%)	4 (3)	2 (2)
Black or African American, n (%)	1 (0)	2 (2)
Asian, n (%)	7 (5)	9 (8)
Other, n (%)	6 (5)	6 (5)

Note

* Round 1: United States (n = 52), United Kingdom (n = 16), Canada (n = 9), the Netherlands (n = 8), Germany (n = 7), Australia (n = 6), Austria (n = 4), Switzerland (n = 3), Norway (n = 3), Ukraine (n = 3), Brazil (n = 2), Denmark (n = 2), France (n = 2), Finland (n = 2), Belgium (n = 1), Croatia (n = 1), Chile (n = 1), Italy (n = 1), Japan (n = 1), Nigeria (n = 1), Peru (n = 1), Philippines (n = 1), Portugal (n = 1), Romania (n = 1), Spain (n = 1) and Thailand (n = 1).

Round 2: United States (n = 50), United Kingdom (n = 17), Australia (n = 7), Canada (n = 6), the Netherlands (n = 8), Norway (n = 5), Germany (n = 3), Denmark (n = 3), France (n = 2), Romania (n = 2), Switzerland (n = 2), Austria (n = 1), Belgium (n = 1), China (n = 1), Croatia (n = 1), Japan (n = 1), Malaysia (n = 1), Peru (n = 1), Philippines (n = 1) and Thailand (n = 1).

All 131 participants who completed the round 1 survey were invited to complete the second round Delphi survey. Of the participants who started the round 2 survey, 95% (n = 114/120) completed it. Similar to round 1, the majority, 59% (n = 67), were female. Overall, 18 countries were represented in this round (see Table 1 for details). A total of 50% (57/114) of participants selected multiple roles (see Appendix S3 to view the roles of participants in the second Delphi round).

### 3.2 Survey results

The percentage of participants who rated each criterion desirable or essential in round 1 and round 2 is shown in Appendix S3. In round 1, participants provided 763 general comments and the following specific suggestions: reword (n = 38), reorder (n = 27), add criteria (n = 23), merge criteria (n = 20), delete criteria (n = 4) and split criteria (n = 1). In round 2, participants provided 91 general comments and the following specific suggestions: merge criteria (n = 7), reword criteria (n = 3) and delete criteria (n = 3). Of the 48 criteria in round 1, 16 were removed, and 7 criteria were merged into 2 criteria. Thus, the round 2 survey had 27 criteria. The changes made after round 1 are described in Appendix S4, and the results of the survey are presented per phase, with each round described separately.

### 3.3 Phase 1: Define process and scope

#### 3.3.1 Round 1 (5 steps)

Between 70% and 95% of participants rated the 21 criteria in Phase 1 as desirable or essential. Qualitative feedback suggested rewording each of the three criteria in step 1 to specify the population and subpopulations, the reasonably relevant options for the decision (including no intervention if applicable), the outcomes and the patient concerns. Participants felt that the four criteria on the evidence summarization process in step 2 were redundant. We therefore merged them into one criterion for the round 2 survey. Similarly, participants felt that the four criteria in step 4, each listing a different stakeholder group that should be part of a team that develops a PDA, should be merged into one criterion. Participant feedback in step 3 suggested spelling out the COI abbreviation, and adding a fifth criterion to report all current and potential conflicts of interest. The general comments also led to the reordering of step 3 and step 4 for the round 2 survey. Also, the following three criteria in step 5 were rated as essential or desirable by 80% or less of participants: (i) there is a systematic process to reduce bias in the definition of the population for the PDA; (ii) there is a systematic process to reduce bias in the definition of the options for the PDA; and (iii) there is a systematic process to reduce bias in the definition of the outcomes or patient concerns for the PDA. The ratings and qualitative feedback led to the removal of step 5 in the round 2 survey. Lastly, participants suggested adding examples to clarify many of the criteria in each step of Phase 1. We added the following example for the first criteria in step 1: ‘*For example, for early stage breast cancer surgery, you will select and summarize evidence about women aged 18 or older with early stage breast cancer stages 1 to 3A If the subpopulation of interest is women over 70 years old or African American women, you will synthesize evidence related to early stage breast cancer surgery for this group, if it is available’*.

#### 3.3.2 Round 2 (4 steps)

Between 86% and 100% of participants rated the ten criteria in Phase 1 as desirable or essential.

Based on qualitative feedback, the criteria in step 3 (‘Assemble the team’) should include a larger stakeholder group to ensure multiple voices prevent the development of a biased PDA. General comments in step 4 (‘Manage conflicts of interest’) explained how PDAs should report a clear, succinct conflict of interest statement. For research teams that develop a suite of PDAs, it may be more practical to have a blanket conflict of interest statement or have the statement be available upon request from the authors. Participants also suggested merging the five criteria in step 4, so that it reads: ‘collect, manage, monitor, and report conflicts of interest’ to avoid repetition.

### 3.4 Phase 2: Finding and appraising evidence

#### 3.4.1 Round 1 (3 steps)

Between 79% and 94% of participants rated the 12 criteria in Phase 2 as desirable or essential. The majority of the participant qualitative feedback focused on rewording the criteria in steps 1 and 2 to be more concise. The five criteria in step 3 (‘Appraise the evidence’) were considered redundant and, based on feedback, were merged into two criteria for the round 2 survey: (a) critically appraise for risk of bias at study level, and (b) critically appraise quality or certainty of the body of evidence. Examples, where suggested by participants, were inserted to help clarify criteria. For instance, participants wanted an example of how to account for risk of bias in study design, so we added the following to the second criteria in step 7: *For example, you may use The Cochrane Risk of Bias Tool*.

#### 3.4.2 Round 2 (3 steps)

Between 86% and 100% of participants rated the nine criteria in Phase 2 as desirable or essential. To avoid redundancy, participants suggested merging two of the criteria in step 2 so that it reads: ‘Systematically select evidence about benefits and harms of each option’. Overall, the qualitative feedback indicated that participants wanted more detail on the hierarchy, grading and sources of evidence. Evidence embedded in PDAs should be derived from systematic reviews. GRADE should be followed to rate the quality of evidence as determined by systematic reviews. The certainty or quality of the evidence should also be critically appraised. Grey literature and social media sources should be avoided unless patient‐relevant harms and benefits and concerns are not sufficiently covered in biomedical databases of published literature.

### 3.5 Phase 3: Presenting evidence and evidence summarization process

#### 3.5.1 Round 1 (4 steps)

Between 74% and 98% of participants rated the 14 criteria in Phase 3 as desirable or essential. The majority of the qualitative comments focused on the redundancy of the criteria in this phase. We therefore reworded the first four criteria in step 1 to be more concise, and we removed the last two criteria for the round 2 survey to avoid redundancy. Furthermore, the three criteria in step 2 (‘Manage COI’) were considered by participants to be too similar to the criteria in step 3 of Phase 1, so we removed them for the round 2 survey. Based on participant feedback, two of the four criteria in step 3 (‘Report’) were removed to avoid redundancy, and the remaining criteria were reworded for the round 2 survey: (a) report the methods used to represent the evidence, and (b) report the evidence summarization process publicly and in a way that is easy to understand. Examples, where suggested by participants, were inserted to help clarify criteria. For instance, participants wanted some guidance on how to present evidence in a way that is easy to understand, so we provided an example in the third criteria of step 8: *For example, the IPDAS chapter on communicating evidence may be used*.

#### 3.5.2 Round 2 (3 steps)

Between 84% and 96% of participants rated the seven criteria in Phase 3 as desirable or essential. Based on qualitative feedback, participants wanted more information in step 1 on *how* to present risk information in a balanced fashion considering the difficulty of this task. PDAs should use absolute risks instead of relative risks. Also, participants sought more detail on the external group in step 3. The criteria should be explicit on the make‐up of the external group, which should include patients to assess how to best present the evidence.

### 3.6 Phase 4: Post‐publication update

#### 3.6.1 Round 1 (1 step)

Step 1 (‘Update’) had one criterion that was rated desirable or essential by 90% of participants: the PDA content is updated when new evidence becomes available. Based on qualitative feedback, the step 1 title was reworded to ‘*Update the evidence’* and the criterion was reworded for the round 2 survey so that it reads: ‘specify process for updates when new evidence becomes available’. Participants suggested that we add an example to the final step and criteria to clarify what is meant by ‘updating the evidence’. We added the following: *For example, the evidence will be updated every year or whenever new relevant evidence is published*.

#### 3.6.2 Round 2 (1 step)

The criterion was rated as desirable or essential by 90% of participants. Qualitative feedback indicated that participants were concerned about the practicality of updating the PDA every time new evidence becomes available. Updating the evidence is a time‐ and resource‐intensive process that requires funding. Guidelines should also be developed to identify *when* and *who* updates the evidence. Relatedly, to increase feasibility, participants felt that a benchmark should be established to update the evidence of PDAs (eg updates are required every three years). Participants also suggested including publication dates on PDAs.

### 3.7 Final process

Overall, the process for selecting and summarizing evidence for PDAs went from five phases consisting of 13 steps and 48 criteria (Round 1) to four phases consisting of 11 steps and 31 criteria (Round 2). Oversight at the beginning of the Delphi process led to the omission of a criterion about the systematic search for the evidence about the effects on the outcomes for each option in the PDA. This oversight was only realized after the completion of the second round of the Delphi survey. At the conclusion of the Delphi process, this criterion was added based on unanimous consensus by the steering group members. An editorial process was undertaken by the steering group to summarize the evidence summarization process for an external audience. The brief, easy‐to‐read summary is presented in Table 2 (see Appendix S5 to view the full evidence summarization process).

**TABLE 2:** Criteria for patient decision aid evidence selection and summarization (see Appendix S4 for details)

Steps	Criteria
Define the decision	Specify the relevant population and/or subpopulations. Specify the relevant reasonable options for the decision, and if applicable, include no treatment or testing. Specify relevant outcomes and patient concerns for this decision.
Report processes and policies	Make public the proposed evidence summarization process in easy‐to‐understand language. Make public the conflict of interest policy.
Assemble editorial team	Assemble a multistakeholder team (eg patients, relevant clinicians, evidence/methodological and other experts).
Manage editorial team conflicts of interest	Collect and update conflicts of interest. Manage conflicts of interest. Report conflicts of interest.
Systematic search	Search for evidence about the appropriateness of options to include. Search for evidence about patient concerns relevant to the options. (Patient‐centred outcomes and concerns may require additional searches and/or research). Search for evidence about the effects of the options on the relevant outcomes (including likelihood of harms and benefits). If personal risk calculation occurs in the aid, systematically search for evidence about predictive factors.
Systematic selection and appraisal	Select and appraise the evidence about the relevant outcomes (including the likelihood of harms and benefits). Select and appraise the evidence about patient concerns relevant to the options. Identify evidence gaps. If personal risk calculation occurs in the aid, select and appraise evidence about predictive factors. Assess evidence uncertainty, and risk of bias in study design, analysis and reporting.
Present the information	Present the evidence for benefit (or evidence gaps) in a balanced way. Present the evidence for harms (or evidence gaps) in a balanced way. Present evidence in easy‐to‐understand formats, following the best practice principles. Present the certainty of the evidence in a way that is easy to understand. Present the evidence in plain language.*
External review	Describe the method of seeking external review by relevant stakeholders.
Update process	Describe the update plan (search, selection, appraisal of new evidence).

* Methods to translate from one language to another are not covered and require further work.

## 4 DISCUSSION

### 4.1 Principal findings

Based on two rounds of a modified Delphi survey, we have developed a process for selecting and summarizing evidence for PDAs that now consists of four phases, 11 steps and 31 criteria. Based on the stakeholders’ feedback, the number of steps was reduced from 13 to 11, and the criteria from 48 to 31 by merging or deleting redundant criteria. The qualitative feedback also informed the rewording of criteria and the addition of examples to improve the clarity of the process for PDA developers. The final 31 criteria were rated as desirable or essential by 84% to 100% of participants.

### 4.2 Strengths and limitations

Our use of the Delphi method to obtain feedback from a multidisciplinary stakeholder group to develop the PDA evidence summarization process is a strength, and the use of this method is supported when evidence is weak or uncertain. There is a dearth of empirical evidence for an evidence summarization process specific to PDA development. This group included patients in each round of the Delphi survey, ensuring that the patient perspective is included in the evidence summarization process. We believe that a major strength of our evidence summarization process is that it encourages the involvement of patients throughout the process and can be used to develop tools for all patient populations, including those who are underrepresented. In terms of limitations, the high attrition rate in the Round 1 survey decreased the size of our sample and may have led to a non‐response bias. Also, we do not know the total number of participants invited to the Round 1 survey because we are unable to determine the total number of potential participants from each listserv. The consistent ‘desirable or essential’ ratings suggest a ceiling effect, which could be reduced in future Delphi studies by substituting a 4‐point scale with a 5‐point scale that includes 3 positive and 2 negative choices.^28^, ^29^ The evidence summarization process has also yet to be piloted. Lastly, the omission of a criterion from the Delphi process prevented participants from providing feedback on whether it is desirable or essential to the evidence summarization process.

### 4.3 Results in context

Our study addresses a significant gap in PDA development guidance by presenting the first process for selecting, summarizing and reporting evidence in PDAs. Prior to the development of this process, the following five items in the IPDAS checklist were the only source of guidance for the selection and summarization of evidence to be included within PDAs: the PDA provides citations to the studies selected, describes how research evidence was selected or synthesized, provides a production or publication date, provides information about the proposed update policy, and describes the quality of the research evidence used.^9^ However, as highlighted by Zadro *et al*, further guidance is needed on how to present evidence, and how to communicate the uncertainty of evidence.^30^, ^31^ For instance, presenting evidence in a ‘balanced’ way can be troublesome in situations where there is evidence of much more benefit than harm or vice versa. Often, options have very little evidence of harms or benefits, so presenting evidence in a ‘balanced’ fashion is not feasible. This is an issue that needs further clarification. The process we have developed with the input of a multidisciplinary stakeholder group provides guidance about criteria to consider when including evidence in a PDA.

### 4.4 Implications

Without a transparent and articulated process for summarizing and presenting evidence, PDAs may not contain an accurate and up‐to‐date summary of the appropriate evidence or present information about evidence quality and certainty. This has implications on the decision‐making process, as the evidence that is included in PDAs can influence the decisions that patients make. Having a process in place may help address frequent concerns from clinicians that important information is missing from PDAs and that numerical data are poorly presented.^32^ Our evidence summarization process addresses these concerns by providing guidance about what should be involved in the process for selecting, summarizing and presenting evidence in PDAs. Future research should involve the piloting of this process with PDA developers and the exploration of how this process can be linked with IPDAS to improve the content and development of PDAs.

## 5 CONCLUSION

Ratings and qualitative feedback from over 100 multidisciplinary stakeholders across 28 countries in two Delphi survey rounds led to the development of a set of criteria for selecting, summarizing, reporting and updating evidence in PDAs. PDAs are promoted as tools that provide evidence‐based, trustworthy information. Widespread adherence to the proposed criteria will help ensure that these tools fulfil that promise.

## CONFLICTS OF INTEREST

Professor Glyn Elwyn has edited and published books that provide royalties on sales by the publishers: the books include *Shared Decision Making* (Oxford University Press) and *Groups* (Radcliffe Press). Dr Elwyn's academic interests are focused on shared decision making and coproduction. He owns copyright in measures of shared decision making and care integration, namely **collabo**RATE **and integ**RATE (measure of care integration), **conside**RATE (patient experience of care in serious illness), **coope**RATE (measure of goal setting), **tole**RATE (clinician attitude to shared decision making), and Observer OPTION‐5 and Observer OPTION‐12 (observer measures of shared decision making). He has in the past provided consultancy for organizations, including the following: (a) Emmi Solutions, LLC who developed patient decision support tools; (b) National Quality Forum on the certification of decision support tools; (c) Washington State Health Department on the certification of decision support tools; and (d) SciMentum LLC, Amsterdam (workshops for shared decision making). He is the founder and director of &think LLC, which owns the registered trademark for Option Grids™ patient decision aids; and founder and director of **SHARP** NETWORK LLC, a provider of training for shared decision making. He provides advice in the domain of shared decision making and patient decision aids to: (a) Access Community Health Network, Chicago (Adviser to Federally Qualified Medical Centers); (b) EBSCO Health for Option Grids ^TM^ patient decision aids (Consultant); (c) Bind On‐Demand Health Insurance (Consultant), (d) PatientWisdom, Inc (Adviser); and (e) Abridge AI Inc (Chief Clinical Research Scientist). Marie‐Anne Durand receives consulting income from EBSCO Health and may receive royalties in the future. She was also a consultant for ACCESS Community Health Network until 2019. All other authors have no conflicts of interest to declare.

## AUTHORS’ CONTRIBUTION

CHS, MD, AMCG, BSA, TH, LPP, MAD and GE initiated the study. CHS, MD and PS created the surveys and facilitated recruitment. PS analysed the data and wrote the first draft of the manuscript. All authors contributed to the writing of the manuscript and approved the final version.

## Supporting information

Appendix S1

Appendix S2

Appendix S3

Appendix S4

Appendix S5

## Data Availability

The data sets used and/or analysed during the current study are available from the corresponding author on reasonable request.

## References

[1] Stacey D, Légaré F, Lewis K, et al. Decision aids for people facing health treatment or screening decisions. Cochrane Database Syst Rev. 2017;4:CD001431. 28402085 10.1002/14651858.CD001431.pub5 PMC6478132

[2] Elwyn G, O'Connor A, Stacey D, et al. International patient decision aids standards (IPDAS) collaboration. Developing a quality criteria framework for patient decision aids: online international Delphi consensus process. BMJ. 2006;333:417‐423. 16908462 10.1136/bmj.38926.629329.AE PMC1553508

[3] Schubbe D, Scalia P, Yen RW, et al. Using pictures to convey health information: A systematic review and meta‐analysis of the effects on patient and consumer health behaviors and outcomes. Patient Educ Couns. 2020;103:1935‐1960. 32466864 10.1016/j.pec.2020.04.010

[4] Scalia P, Durand MA, Forcino RC, et al. Implementation of the uterine fibroids Option Grid patient decision aids across five organizational settings: a randomized stepped‐wedge study protocol. Implement Sci. 2019;14:88. 31477140 10.1186/s13012-019-0933-z PMC6721118

[5] Durand MA, Alam S, Grande SW, Elwyn G. ‘Much clearer with pictures’: using community‐based participatory research to design and test a Picture Option Grid for underserved patients with breast cancer. BMJ open. 2016;6:e010008. 10.1136/bmjopen-2015-010008 PMC4746463 26839014

[6] Yen RW, Durand MA, Harris C, et al. Text‐only and picture conversation aids both supported shared decision making for breast cancer surgery: Analysis from a cluster randomized trial. Patient Educ Couns. 2020;103:2235‐2243. 32782181 10.1016/j.pec.2020.07.015

[7] Scalia P, Durand MA, Faber M, Kremer JA, Song J, Elwyn G. User‐testing an interactive option grid decision aid for prostate cancer screening: lessons to improve usability. BMJ open. 2019;9:e026748. 10.1136/bmjopen-2018-026748 PMC6538002 31133587

[8] Dannenberg MD, Durand MA, Montori VM, Reilly C, Elwyn G. Existing evidence summarization methods cannot guarantee trustworthy patient decision aids. J Clin Epidemiol. 2018;102:69‐77. 29928973 10.1016/j.jclinepi.2018.06.003

[9] Elwyn G, O'Connor AM, Bennett C, et al. Assessing the quality of decision support technologies using the International Patient Decision Aid Standards instrument (IPDASi). PLoS One. 2009;4:e4705. 19259269 10.1371/journal.pone.0004705 PMC2649534

[10] Montori VM, LeBlanc A, Buchholz A, Stilwell DL, Tsapas A. Basing information on comprehensive, critically appraised, and up‐to‐date syntheses of the scientific evidence: a quality dimension of the international patient decision aid standards. BMC Med Inform Decis Mak. 2013;13(Suppl 2):S5. 10.1186/1472-6947-13-S2-S5 PMC4044946 24625191

[11] Durand MA, Witt J, Joseph‐Williams N, et al. Minimum standards for the certification of patient decision support interventions: feasibility and application. Patient Educ Couns. 2015;98:462‐468. 25577469 10.1016/j.pec.2014.12.009

[12] Joseph‐Williams N, Newcombe R, Politi M, et al. Toward minimum standards for certifying patient decision aids: a modified Delphi consensus process. Med Decis Making. 2014;34:699‐710. 23963501 10.1177/0272989X13501721

[13] Hoffmann T, Bakhit M, Durand M, Perestelo‐Pérez L, Saunders C, Brito J. Basing information on comprehensive, critically appraised, and up‐to‐date syntheses of the scientific evidence: an update from the International Patient Decision Aid Standards. (under review). Forthcoming at: http://ipdas.ohri.ca/resources.html 10.1177/0272989X21996622 33660539

[14] Washington Health Care Authority . Patient decision aids (PDAs). 2018 https://www.hca.wa.gov/about‐hca/healthier‐washington/patient‐decision‐aids‐pdas

[15] Higgins JPT, Green S. Cochrane handbook for systematic reviews of interventions version 5.0. York: The Cochrane Collaboration. 2009.

[16] Brożek J, Guyatt GGRADE. Handbook for grading quality of evidence and strength of recommendations. The GRADE Working Group; 2013.

[17] Qaseem A, Forland F, Macbeth F, Ollenschlager G, Phillips S. van der Wees P, Board of Trustees of the Guidelines International Network. Guidelines International Network: toward international standards for clinical practice guidelines. Ann Intern Med. 2012;156:525‐531. 22473437 10.7326/0003-4819-156-7-201204030-00009

[18] National Academy of Sciences . Standards for systematic reviews. 2011 http://www.nationalacademies.org/hmd/Reports/2011/FindingWhat‐Works‐in‐Health‐Care‐Standards‐for‐Systematic‐Reviews/Standards.aspx

[19] Moher D, Liberati A, Tetzlaff J. Altman DG for the PRISMA group. Preferred reporting items for systematic reviews and meta‐analyses: the PRISMA statement. PLoS Med. 2009;6:e1000097. 19621072 10.1371/journal.pmed.1000097 PMC2707599

[20] Brouwers MC, Kho ME, Browman GP, et al. AGREE Next Steps Consortium. The Global Rating Scale complements the AGREE II in advancing the quality of practice guidelines. J Clin Epidemiol. 2012;65:526‐534. 22189163 10.1016/j.jclinepi.2011.10.008

[21] Brouwers MC, Kho ME, Browman GP, et al. AGREE Next Steps Consortium. Development of the AGREE II, part 1: performance, usefulness and areas for improvement. CMAJ. 2010;182:1045‐1052. 20513780 10.1503/cmaj.091714 PMC2900328

[22] Brouwers MC, Kho ME, Browman GP, et al. AGREE Next Steps Consortium. Development of the AGREE II, part 2: assessment of validity of items and tools to support application. CMAJ. 2010;182:E472‐E478. 20513779 10.1503/cmaj.091716 PMC2900368

[23] Cluzeau F. AGREE Research Trust. Conflicting recommendations. Let's not forget AGREE. BMJ. 2009;338:b407. 19188229 10.1136/bmj.b407

[24] Lawani MA, Valéra B, Fortier‐Brochu É, et al. Five shared decision making tools in 5 months: use of rapid reviews to develop decision boxes for seniors living with dementia and their caregivers. Syst Rev. 2017;6:56. 28298241 10.1186/s13643-017-0446-2 PMC5353791

[25] Durand MA, Dannenberg MD, Saunders CH, et al. Modified Delphi survey for the evidence summarisation of patient decision aids: Study protocol. BMJ Open. 2019;9:e026701. 10.1136/bmjopen-2018-026701 PMC6475258 30904876

[26] Murphy MK, Black NA, Lamping DL, et al. Consensus development methods, and their use in clinical guideline development. Health Technol Assess. 1998;2:1‐88. 9561895

[27] *Standards for Guideline Development* . U.S. Preventive Services Task Force. June 2018.

[28] Oostendorp LJ, Durand MA, Lloyd A, et al. Measuring organisational readiness for patient engagement (MORE): an international online Delphi consensus study. BMC Health Serv Res. 2015;15:61. 25879457 10.1186/s12913-015-0717-3 PMC4334597

[29] Moret L, Nguyen JM, Pillet N, Falissard B, Lombrail P, Gasquet I. Improvement of psychometric properties of a scale measuring inpatient satisfaction with care: a better response rate and a reduction of the ceiling effect. BMC Health Serv Res. 2007;7:197. 18053170 10.1186/1472-6963-7-197 PMC2225402

[30] Zadro JR, Traeger AC, Décary S, O'Keeffe M. Problem with patient decision aids. BMJ. Evid Based Med. 2020;bmjebm‐2020‐111371. 10.1136/bmjebm-2020-111371 32439722

[31] Montori VM, LeBlanc A, Buchholz A, Stilwell DL, Tsapas A. Basing information on comprehensive, critically appraised, and up‐to‐date syntheses of the scientific evidence: a quality dimension of the International Patient Decision Aid Standards. BMC Med Inform Decis Mak. 2013;13:S5. 10.1186/1472-6947-13-S2-S5 PMC4044946 24625191

[32] Scalia P, Durand MA, Berkowitz JL, et al. The impact and utility of encounter patient decision aids: systematic review, meta‐analysis and narrative synthesis. Patient Educ Couns. 2019;102:817‐841. 30612829 10.1016/j.pec.2018.12.020

