# Supplementary material for: Processes for evidence summarization for patient decision aids: A Delphi consensus study
Source: Health Expect. 2021 May 15;24(4):1178–86. doi: 10.1111/hex.13244 (PMC8369090; doi:10.1111/hex.13244)
Supplement: Supplementary file 1 — Appendix S1 [file HEX-24-1178-s002.docx]

Appendix 1. List of 48 criteria sent to participants for round 1 of the Delphi study.

| **Topic to be covered in the evidence summarization process** | **Phase** | **Step** | **Criteria** |
| --- | --- | --- | --- |
| Establishing transparency | Phase I: Define Process and Scope | Define the question | The question is defined according to which population is relevant for this PDA. |
|  |  |  | The question is defined according to which options are relevant for this PDA. |
|  |  |  | The question is defined according to which outcomes or patient concerns are relevant for this PDA. |
|  |  | Document process and policies | The evidence summarization process is documented. |
|  |  |  | The evidence summarization process minimizes bias. |
|  |  |  | The evidence summarization process minimizes conflicts of interest. |
|  |  |  | The conflict of interest policy applying to people who summarize evidence is documented. |
| Management of conflict of interest |  | Manage COI | The conflicts of interest of people who summarize evidence are collected. |
|  |  |  | Actions are taken to manage relevant conflicts of interest. |
|  |  |  | The actions taken on relevant conflicts of interest are documented. |
|  |  |  | Conflicts of interest are monitored over the course of PDA development. |
| Guideline development group composition |  | Assemble team | A multidisciplinary team is assembled. |
|  |  |  | The team comprises clinicians. |
|  |  |  | The team comprises methodological experts. |
|  |  |  | The team comprises patient or consumer representatives. |
|  |  | Define the scope of patient decision aid content | The population for whom the PDA is designed for is appropriate. |
|  |  |  | There is a systematic process to reduce bias in the definition of the population for the PDA. |
|  |  |  | The options for inclusion in the PDA are appropriate for the intended population. |
|  |  |  | There is a systematic process to reduce bias in the definition of the options for the PDA. |
|  |  |  | The outcomes or patient concerns for inclusion in the PDA are appropriate for the intended population and options. |
|  |  |  | There is a systematic process to reduce bias in the definition of the outcomes or patient concerns for the PDA. |
| Guideline and systematic review intersection | PHASE II: Finding & Appraising Evidence | Search for evidence | There is a systematic search for evidence that relates to the options included in the PDA. |
|  |  |  | There is a systematic search for evidence that relates to the outcomes or patient concerns included in the PDA. |
|  |  |  | If the PDA is customizable to individual patient factors, there is a systematic search for evidence of how individual patient factors influence the expected outcomes. |
| Establishing evidence foundations and rating strength of recommendation |  | Select evidence | There is a systematic process for selecting evidence for outcomes or patient concerns to include in the PDA (where evidence is not available, can directly ask patients). |
|  |  |  | There is a systematic process for selecting evidence (or evidentiary gaps) about potential benefits relevant to each option. |
|  |  |  | There is a systematic process for selecting evidence (or evidentiary gaps) about potential harms relevant to each option. |
|  |  |  | If the PDA is customizable to individual patient factors, there is a systematic process for selecting relevant risk predictors to include in the PDA. |
|  |  | Appraise evidence | Evidence selected for inclusion in the PDA is critically appraised with a defined protocol (such as GRADE). |
|  |  |  | The protocol for critical appraisal of evidence accounts for risks of bias in study design. |
|  |  |  | The protocol for critical appraisal of evidence accounts for risks of bias in study analysis and reporting. |
|  |  |  | The protocol for critical appraisal of evidence accounts for assessment of certainty of evidence with attention to risk of bias, precision, directness, consistency, and publication bias. |
|  |  |  | The conflicts of interest of study authors related to selected evidence is appraised. |
| Articulation of information | PHASE III: Presenting Evidence | Articulate the information | The evidence (or evidentiary gaps) about potential benefits relevant to each option is summarized in balanced ways, not expected to bias the interpretation. |
|  |  |  | The evidence (or evidentiary gaps) about potential harms relevant to each option is summarized in balanced ways, not expected to bias the interpretation. |
|  |  |  | The evidence (or evidentiary gaps) is summarized in ways that are easy to understand. |
|  |  |  | The certainty of the evidence is described in ways that are easy to understand. |
|  |  |  | The evidence summarization process is described in ways that are easy to understand. |
|  |  |  | The funding used to summarize the evidence (and develop the PDA) is reported. |
|  |  | Manage COI | The conflicts of interest of people who summarize evidence are collected again before publishing the PDA. |
|  |  |  | Any change to the conflicts of interest of people who summarize evidence are reported. |
|  |  |  | Actions are taken to manage relevant conflicts of interest. |
|  |  | Report | The methods used to translate evidence to risk communication formats are reported. |
|  |  |  | The approach to readability of summarized evidence is reported. |
|  |  |  | The summarization process is reported publicly. |
|  |  |  | The conflict of interest of people who summarize evidence are reported publicly. |
|  |  | Review | The PDA is reviewed externally. |
| Updating | PHASE IV: Post- publication update | Update | The PDA content is updated when new evidence becomes available. |
