# Supplementary material for: Processes for evidence summarization for patient decision aids: A Delphi consensus study
Source: Health Expect. 2021 May 15;24(4):1178–86. doi: 10.1111/hex.13244 (PMC8369090; doi:10.1111/hex.13244)
Supplement: Supplementary file 2 — Appendix S2 [file HEX-24-1178-s003.docx]

Appendix 2. The survey invitation.

SUBJ: Help us make more trustworthy patient materials: provide your feedback through a survey

To the members of [group name/list-serv name] –

We are an international workgroup, led by [blinded for review] and [blinded for review] at [blinded for review]. We noticed a need for more clarity about how to select and summarize the evidence included in patient decision aids. Patient decision aids influence the decisions that patients make - so the need for trustworthy tools is important.

We wish to have your perspective, as an expert, patient, or other stakeholder.

**Please could you provide feedback via 2-3 surveys over the next few weeks?** Each survey should take less than 25 minutes.

Please click the link below for more information and the first survey.

Many thanks,

The Evidence Summarization workgroup
