# Supplementary material for: Processes for evidence summarization for patient decision aids: A Delphi consensus study
Source: Health Expect. 2021 May 15;24(4):1178–86. doi: 10.1111/hex.13244 (PMC8369090; doi:10.1111/hex.13244)
Supplement: Supplementary file 3 — Appendix S3 [file HEX-24-1178-s005.docx]

Appendix 3. The roles of participants in each round of the Delphi survey.

| **Round 1** |  |
| --- | --- |
| **Role(s)** | **N** |
| Researcher | 21 |
| Clinician | 20 |
| Patient | 8 |
| Other | 8 |
| Policymaker | 7 |
| PDA Developer | 2 |
| IPDAS Collaboration Member | 1 |
| Researcher and Clinician | 16 |
| Researcher & PDA Developer | 6 |
| Researcher & Other | 5 |
| Patient & Other | 4 |
| Researcher & Patient | 3 |
| Researcher & IPDAS Collaboration Member | 3 |
| Researcher & Policymaker | 2 |
| Clinician & Other | 2 |
| PDA Developer & Other | 2 |
| PDA Developer & Policymaker | 1 |
| Researcher, PDA Developer & IPDAS Collaboration Member | 5 |
| Researcher, PDA Developer, Clinician | 3 |
| Researcher, Clinician, Other | 2 |
| Researcher, PDA Developer, Other | 1 |
| Researcher, IPDAS Collab Member, Patient | 1 |
| Policymaker, Patient, Clinician | 1 |
| Researcher, Policymaker, Other | 1 |
| PDA Developer, Clinician, Other | 1 |
| PDA Developer, Researcher, Patient | 1 |
| Patient, Clinician, Other | 1 |
| PDA developer, IPDAS collaboration member, Clinician | 1 |
| PDA Developer, Researcher, policymaker and clinician | 1 |
| PDA Developer, Researcher, patient and clinician | 1 |

| **Round 2** |  |
| --- | --- |
| **Role(s)** | **N** |
| Researcher | 24 |
| Patient | 13 |
| Clinician | 10 |
| Other | 9 |
| PDA Developer | 1 |
| Policymaker | 1 |
| Researcher & Clinician | 11 |
| Researcher & PDA Developer | 7 |
| Patient & Other | 5 |
| Researcher & IPDAS Collaboration Member | 4 |
| Clinician & Other | 4 |
| Researcher & Policymaker | 3 |
| Researcher & Other | 1 |
| PDA Developer & Policymaker | 1 |
| IPDAS Collaboration Member & Clinician | 1 |
| PDA Developer, Researcher, Clinician | 6 |
| Researcher, Clinician, Other | 2 |
| PDA Developer, Researcher, Other | 2 |
| Researcher, Policymaker, Clinician | 1 |
| PDA Developer, Researcher, Patient | 1 |
| PDA Developer, Researcher, Policymaker | 1 |
| PDA Developer, Researcher, IPDAS Collab Member, Other | 1 |
| PDA Developer, Researcher, IPDAS Collab Member, Policymaker | 1 |
| PDA Developer, Researcher, IPDAS Collab Member, Clinician | 1 |
| PDA Developer, Researcher, Patient, Other | 1 |
| PDA Developer, Researcher, Patient, Clinician | 1 |
| PDA Developer, Researcher, Policymaker, Patient, Clinician | 1 |
