# Supplementary material for: Processes for evidence summarization for patient decision aids: A Delphi consensus study
Source: Health Expect. 2021 May 15;24(4):1178–86. doi: 10.1111/hex.13244 (PMC8369090; doi:10.1111/hex.13244)
Supplement: Supplementary file 4 — Appendix S4 [file HEX-24-1178-s001.docx]

Appendix 4. Participants’ ratings of the proposed criterion.

| **Round 1** | | | **Changes made after Round 1 feedback** | **Round 2** | | |
| --- | --- | --- | --- | --- | --- | --- |
|  | **Criteria** | **% who rated the criterion desirable or essential** |  |  | **Criteria** | **% who rated the item desirable or essential** |
| **Phase I** | | |  | **Phase I** | | |
| Step 1: Define the question | The question is defined according to which population is  relevant for this PDA. | 88 | Reworded | Step 1: Define the clinical decision and outcomes | Specify the population and subpopulations relevant for this decision. | 98 |
|  | The question is defined according to which options are  relevant for this PDA. | 87 | Reworded |  | Specify all reasonably relevant options for this decision, including no intervention if applicable. | 100 |
|  | The question is defined according to which outcomes or  patient concerns are relevant for this PDA. | 94 | Reworded |  | Specify all relevant outcomes and patient concerns for this decision. | 97 |
| Step 2: Document process and policies | The evidence summarization process is documented. | 93 | Reworded | Step 2: Specify the process and policies for evidence summarization | Report the evidence summarization process publicly. | 90 |
|  | The evidence summarization process minimizes bias. | 87 | Removed |  | - | - |
|  | The evidence summarization process minimizes conflicts of  interest. | 79 | Removed |  | - | - |
|  | The conflict of interest policy applying to people who summarize  evidence is documented. | 87 | Removed |  | - | - |
| Step 3: Manage COI | The conflicts of interest of people who summarize evidence are collected. | 95 | All criteria in step 3 are reworded and moved to step 4. Criteria to report relevant COI is added. | Step 3: Assemble the team | Assemble a multidisciplinary team with relevant stakeholders including patients, clinicians, and methodological experts. | 92 |
|  | Actions are taken to manage relevant conflicts of interest. | 88 |  |  | - | - |
|  | The actions taken on relevant conflicts of interest are documented. | 84 |  |  | - | - |
|  | Conflicts of interest are monitored over the course of PDA development. | 84 |  |  | - | - |
| Step 4: Assemble team | A multidisciplinary team is assembled. | 94 | All 4 criteria are merged into 1 criteria and moved to step 3 | Step 4: Manage conflicts of interest | Report the conflict of interest policy publicly. | 95 |
|  | The team comprises clinicians. | 94 |  |  | Collect all current and potential conflicts of interest. | 96 |
|  | The team comprises methodological experts | 90 |  |  | Manage relevant conflicts of interest. | 93 |
|  | The team comprises patient or consumer representatives. | 94 |  |  | Report all relevant conflicts of interest. | 96 |
|  | - | - |  |  | Monitor conflicts of interest. | 86 |
| Step 5: Define the scope of patient decision aid content | The population for whom the PDA is designed for is appropriate. | 86 | Removed |  | - | - |
|  | There is a systematic process to reduce bias in the definition of the  population for the PDA. | 70 | Removed |  | - | - |
|  | The options for inclusion in the PDA are appropriate for the  intended population. | 87 | Removed |  | - | - |
|  | There is a systematic process to reduce bias in the definition of the  options for the PDA. | 80 | Removed |  | - | - |
|  | The outcomes or patient concerns for inclusion in the PDA are  appropriate for the intended population and options. | 86 | Removed |  | - | - |
|  | There is a systematic process to reduce bias in the definition of the  outcomes or patient concerns for the PDA. | 79 | Removed |  | - | - |
| **Phase II** | | |  | **Phase II** | | |
| Step 1: Search for evidence | There is a systematic search for evidence that relates to the options  included in the PDA. | 88 | Reworded | Step 1: Search for relevant evidence | Systematically search for evidence about the options presented in the patient decision aid. | 98 |
|  | There is a systematic search for evidence that relates to the  outcomes or patient concerns included in the PDA. | 85 | Reworded |  | Systematically search for evidence about the outcomes and patient concerns. | 93 |
|  | If the PDA is customizable to individual patient factors, there is a  systematic search for evidence of how individual patient factors  influence the expected outcomes. | 79 | Reworded |  | If the PDA is customizable to individual patient factors, systematically search for evidence about how individual patient factors influence outcomes. | 92 |
| Step 2: Select evidence | There is a systematic process for selecting evidence for outcomes  or patient concerns to include in the PDA (where evidence is not available, can directly ask patients). | 94 | Reworded | Step 2: Select the relevant evidence | Systematically select evidence about outcomes or patient concerns; ask patients if evidence is not available. | 88 |
|  | There is a systematic process for selecting evidence (or evidentiary gaps) about potential benefits relevant to each option. | 92 | Reworded |  | Systematically select evidence or evidence gaps about benefits of each option. | 98 |
|  | There is a systematic process for selecting evidence (or evidentiary gaps) about potential harms relevant to each option. | 83 | Reworded |  | Systematically select evidence or evidence gaps about the harms of each option. | 100 |
|  | If the PDA is customizable to individual patient factors, there is a  systematic process for selecting relevant risk predictors to include  in the PDA. | 82 | Reworded |  | If the patient decision aid is customizable to individual patient factors, systematically select harm predictors for individual patient factors. | 88 |
| Step 3: Appraise evidence | Evidence selected for inclusion in the PDA is critically appraised  with a defined protocol (such as GRADE). | 93 | Merged with criteria 3 and 4 | Step 3:  Appraise selected evidence | Critically appraise for risk of bias at study level. | 87 |
|  | The protocol for critical appraisal of evidence accounts for risks of bias in study design. | 92 | Reworded |  | Critically appraise quality or certainty of the body of evidence. | 95 |
|  | The protocol for critical appraisal of evidence accounts for risks of bias in study analysis and reporting. | 83 | Merged with criteria 1 and 4 |  | - | - |
|  | The protocol for critical appraisal of evidence accounts for  assessment of certainty of evidence with attention to risk of bias, precision, directness, consistency, and publication bias. | 89 | Merged with criteria 1 and 3 |  | - | - |
|  | The conflicts of interest of study authors related to selected evidence is appraised. | 87 | Removed |  | - | - |
| **Phase III** | | |  | **Phase III** | | |
| Step 1: Articulate the information | The evidence (or evidentiary gaps) about potential benefits relevant to each option is summarized in balanced ways, not expected to  bias the interpretation. | 92 | Reworded | Step 1:  Present the information | Present the evidence or evidentiary gaps about benefits in a balanced way. | 94 |
|  | The evidence (or evidentiary gaps) about potential harms relevant  to each option is summarized in balanced ways, not expected to  bias the interpretation. | 92 | Reworded |  | Present the evidence or evidentiary gaps about harms in a balanced way. | 95 |
|  | The evidence (or evidentiary gaps) is summarized in ways that are easy to understand. | 98 | Reworded |  | Present the evidence or evidentiary gaps in a way that is easy to understand. | 96 |
|  | The certainty of the evidence is described in ways that are easy to understand. | 98 | Reworded |  | Present the quality or certainty of the evidence in a way that is easy to understand. | 93 |
|  | The evidence summarization process is described in ways that are easy to understand. | 83 | Removed |  | - | - |
|  | The funding used to summarize the evidence (and develop the PDA) is reported. | 86 | Removed |  | - | - |
| Step 2: Manage COI | The conflicts of interest of people who summarize evidence are collected again before publishing the PDA. | 76 | Step 2: “Manage COI” is removed | Step 2: Report evidence | Report the methods used to represent the evidence. | 84 |
|  | Any change to the conflicts of interest of people who summarize evidence are reported. | 80 |  |  | Report the evidence summarization process publicly and in a way that is easy to understand. | 89 |
|  | Actions are taken to manage relevant conflicts of interest. | 83 |  |  | - | - |
| Step 3: Report | The methods used to translate evidence to risk communication  formats are reported. | 83 | Criteria 1 and 3 are reworded and moved to step 2. Criteria 2 and 4 are removed. | Step 3: Review evidence | Send the patient decision aid to an external group for review. | 88 |
|  | The approach to readability of summarized evidence is reported. | 74 |  |  | - | - |
|  | The summarization process is reported publicly. | 83 |  |  | - | - |
|  | The conflict of interest of people who summarize evidence are reported publicly. | 89 |  |  | - | - |
| Step 4: Review | The PDA is reviewed externally. | 92 | Reworded and moved to step 3 |  | - | - |
| **Phase IV** | | |  | **Phase IV** | | |
| Step 1: Update | The PDA content is updated when new evidence becomes  available. | 90 | Reworded | Step 1: Update the evidence | Specify process for updates when new evidence becomes available. | 90 |
