# Supplementary material for: Processes for evidence summarization for patient decision aids: A Delphi consensus study
Source: Health Expect. 2021 May 15;24(4):1178–86. doi: 10.1111/hex.13244 (PMC8369090; doi:10.1111/hex.13244)
Supplement: Supplementary file 5 — Appendix S5 [file HEX-24-1178-s004.docx]

Appendix 5. The final process for selecting and summarizing evidence for PDAs.

| **Steps** | **Criteria** |
| --- | --- |
| 1. Define the clinical decision | Specify the population and subpopulations relevant for this decision. *For example, for early stage breast cancer surgery, you will select and summarize evidence about women aged 18 or older with early stage breast cancer stages 1 to 3A. If the subpopulation of interest is women over 70 years old or African American women, you will synthesize evidence related to early stage breast cancer surgery for this group, if it is available.* |
|  | Specify all reasonably relevant options for this decision, including no treatment or testing, if applicable. *For example, for prostate cancer, you will summarize the evidence for active surveillance, radical prostatectomy or radiotherapy.* |
|  | Specify all relevant outcomes and patient concerns for this decision. *For example, for early stage breast cancer surgery, relevant outcomes include survival, recurrence, re-operation etc. Concerns may include costs and other practical information. If absent from the literature, seek feedback from patients via survey, focus groups or other methods to determine which outcomes and concerns are important to patients.* |
| 2. Report the process and policies | Describe the evidence summarization process publicly. *For example, this could be a protocol or report.* |
|  | Report the conflict of interest policy publicly. |
| 3. Assemble the team | Assemble a multidisciplinary team with relevant stakeholders (including patients, relevant clinicians and methodological experts). *For example, the team may include two editors, one clinician with relevant clinical expertise for this decision, 2 patient/patient representatives, 1 researcher specializing in communication in healthcare or another methodological expert.* |
| 4. Manage conflicts of interest from people involved in the editorial process | Collect all current and potential conflicts of interest. |
|  | Manage relevant conflicts of interest. *For example, you may disclose and monitor or prohibit involvement from people with relevant conflicts.* |
|  | Report relevant conflicts of interest |
|  | Update conflicts of interest. *For example, you may ask editors to update their COI disclosure when there is a change.* |
| 5. Search for relevant evidence | Systematically search for evidence about the appropriateness of options to include in the PDA. *For example, the search may include clinical practice guidelines or medical databases such as Medline and Cinahl.* |
|  | Systematically search for evidence about the outcomes (harms and benefits) and patient concerns important to address in the PDA. Gray literature, social media and/or conducting focus groups should be avoided, unless patient relevant harms and benefits and concerns are not sufficiently covered in biomedical databases. In this case, it may be necessary to consider these sources. |
|  | Systematically search for evidence about the effects on (or likelihood of) the outcomes with the options in the PDA. |
|  | If the PDA is customizable to individual patient factors, systematically search for evidence about how individual patient factors influence outcomes. |
| 6. Select relevant evidence | Systematically select evidence about outcomes or patient concerns to address in the PDA. Ask patients about concerns if evidence is not available. |
|  | Systematically select evidence about benefits of each option. When possible, evidence should be selected from systematic reviews. If systematic reviews are not available, evidence from randomized controlled trials (RCTs) should be used. Evidence from appropriate study designs (e.g., cohort and case-control studies) may be appropriate if RCTs are not available. Identify evidence gaps. |
|  | Systematically select evidence about the harms of each option. When possible, evidence should be selected from systematic reviews. If systematic reviews are not available, evidence from randomized controlled trials (RCTs) should be used. Evidence from appropriate study designs (e.g., cohort and case-control studies) may be appropriate if RCTs are not available. Identify evidence gaps. |
|  | If the PDA is customizable to individual patient factors, systematically select evidence about outcome predictors. |
| 7. Appraise selected evidence | Critically appraise the evidence with a defined protocol. |
|  | Account for risk of bias in study design. *For example, you may use The Cochrane Risk of Bias Tool. ^*^* |
|  | Account for risk of bias in study analysis and reporting. |
|  | Account for assessment of the certainty of evidence. *For example, the GRADE domains should be used.^11^* |
| 8. Present the information | Present the evidence (or gaps in the evidence) about benefits in a balanced way (i.e., not expected to bias interpretation). Include patient-reported benefits where possible. |
|  | Present the evidence (or gaps in the evidence) about harms in a balanced way (i.e., not expected to bias interpretation). Include patient-reported benefits where possible. |
|  | Present the evidence (or gaps in the evidence) in a way that is easy to understand. *For example, the IPDAS chapter on communicating evidence may be used. ^4^* |
|  | Present the certainty of the evidence in a way that is easy to understand. |
|  | Describe the evidence summarization process in a way that is easy to understand. *For example, we will use plain language.* |
| 9. Report evidence | Report the methods used to translate the evidence. |
|  | Report the evidence summarization process publicly. |
| 10. Review evidence | Send the PDA to an external group for review. |
| 11. Update the evidence | Update the evidence periodically or continuously. *For example, the evidence will be updated every year or whenever new relevant evidence is published*. |

*Higgins JP, Altman DG, Gøtzsche PC, Jüni P, Moher D, Oxman AD, Savović J, Schulz KF, Weeks L, Sterne JA. The Cochrane Collaboration’s tool for assessing risk of bias in randomised trials. Bmj. 2011 Oct 18;343:d5928.
